# Biosynthesis of isoprenoids, polyunsaturated fatty acids and flavonoids in *Saccharomyces cerevisiae*

**DOI:** 10.1186/1475-2859-5-20

**Published:** 2006-05-23

**Authors:** Joseph A Chemler, Yajun Yan, Mattheos AG Koffas

**Affiliations:** 1 Department of Chemical and Biological Engineering, State University of New York at Buffalo, Buffalo, NY 14260-4200, USA

## Abstract

Industrial biotechnology employs the controlled use of microorganisms for the production of synthetic chemicals or simple biomass that can further be used in a diverse array of applications that span the pharmaceutical, chemical and nutraceutical industries. Recent advances in metagenomics and in the incorporation of entire biosynthetic pathways into *Saccharomyces cerevisiae *have greatly expanded both the fitness and the repertoire of biochemicals that can be synthesized from this popular microorganism. Further, the availability of the *S. cerevisiae *entire genome sequence allows the application of systems biology approaches for improving its enormous biosynthetic potential. In this review, we will describe some of the efforts on using *S. cerevisiae *as a cell factory for the biosynthesis of high-value natural products that belong to the families of isoprenoids, flavonoids and long chain polyunsaturated fatty acids. As natural products are increasingly becoming the center of attention of the pharmaceutical and nutraceutical industries, the use of *S. cerevisiae *for their production is only expected to expand in the future, further allowing the biosynthesis of novel molecular structures with unique properties.

## Introduction

Microbial biosynthesis of natural products is an emerging area of metabolic engineering and industrial biotechnology that offers significant advantages over conventional chemical methods or extraction from biomass. Lower energy requirements, lower CO_2 _emissions, less toxic waste in the form of solvents and metal catalysts, simpler purification schemes, renewable feed stocks such as corn or soybeans, and the general ability of enzymes to perform chiral synthesis are among the improvements to be gained by microbial synthesis.

Within this context, almost 10 years since the publication of its entire genome sequence [1], *Saccharomyces cerevisiae*, remains the key model eukaryotic organism, with several features that make it a useful tool for both industrial applications and research:

1. It grows easily and adapts well in controlled fermentation environments.

2. Powerful tools for genetic engineering have been extensively developed and are readily available [2-4].

3. It is nonpathogenic and has been used since ancient times in food technology.

4. Unlike bacteria that require extensive protein engineering [5,6], yeast can readily express functional type II P450 monooxygenases, required for the biosynthesis of higher eukaryotic secondary metabolites [7,8].

Moreover, the availability of the entire genome sequence has made possible the collection of comprehensive information that allows a systems biology approach towards strain development and optimization [9-11]. As such, *S. cerevisiae *truly appears to be an ideal production platform for important secondary metabolites, such as natural products.

Natural products tend to encompass a wide array of small molecules derived from both eukaryotic (plants, marine organisms) and prokaryotic species. Their chemical structure has been fine-tuned through billions of years of evolution to serve specific functions in living organisms. Many natural products are high-value chemicals, since only small quantities can be isolated from their native biosynthetic organisms with the majority of them utilized as therapeutic agents for (among others) infectious diseases and cancer. The advent of genomics and metagenomics has allowed not only the discovery of a huge number of new promising metabolites, but also the elucidation of their biosynthetic pathways. As a result, the use of well-characterized hosts for their efficient, large scale production is now possible.

Since presenting the entire range of natural product biosynthesis in *S. cerevisiae *would require an extensive amount of space, in the next few paragraphs we will only highlight some of the most characteristic work in this area.

### Isoprenoid biosynthesis

Isoprenoids (also known as terpenoids) belong to a vast group of secondary metabolites [12] that include carotenoids, sterols, polyprenyl alcohols, ubiquinone (coenzyme Q), heme A and prenylated proteins. They are of valuable commercial interest as food colorants and antioxidants (carotenoids), aroma and flavor enhancers (terpenes), nutraceuticals (ubiquinone), and antiparasitic and anticarcinogenic compounds (taxol) [13-15].

**Figure 1:**
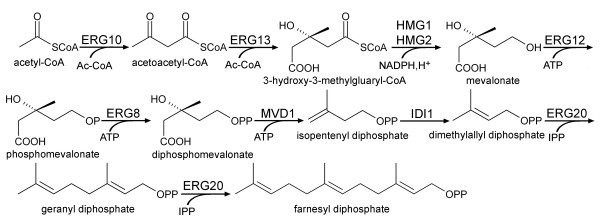
**Biosynthesis of isoprenoids by the mevalonate pathway**. IPP: isopentenyl pyrophosphate; *ERG10*: acetoacetyl-CoA ligase; *ERG13*: 2-hydroxy-3-methylglutaryl-CoA synthase (HMG-CoA synthase); *HMG1 *and *HMG2*: 2-hydroxy-3-methylglutaryl-CoA reductases; *ERG12*: mevalonate kinase; *ERG8*: phosphomevalonate kinase; *MVD1*: diphosphomevalonate decarboxylase; *IDI1*: isopentenyl pyrophosphate:dimethylallyl diphosphate isomerase; *ERG20*: farnesyl diphosphate synthase.

All isoprenoids are synthesized from a universal compound called isopentenyl diphosphate (IPP). The first pathway to IPP, the mevalonic acid (MVA) pathway, was first described by Bloch and Lynen [16,17]. In yeast, the mevalonate pathway (Figure 1) is chiefly employed to form ergosterol (provitamin D_2_) which is an essential part of the yeast membrane and provides membrane permeability and fluidity [18-21].

Some of the most important isoprenoids and their engineered biosynthesis in *S. cerevisiae *will be further described.

#### Taxol

Taxol is a complex substituted terpenoid that was first isolated from the bark of Pacific yew (*Taxus brevifolia*). It is a potent anticancer agent that has been approved for the treatment of refractory ovarian and metastatic breast cancer. Currently, the demand for taxol greatly exceeds the supply that can be isolated from its natural sources [22]. Very elegant total chemical syntheses of taxol have been developed but, because of low yields and high costs, none of these approaches is suitable for its commercial production. Similarly, *Taxus *cell cultures have yet to reach commercialization due to low and unstable productivity [23].

With the partial elucidation of the taxol biosynthetic pathway the possibility of taxadiene (a taxol biosynthetic intermediate) biosynthesis in *Escherichia coli *was successfully demonstrated [24]. One major limitation with that approach however is that the prokaryotic *E. coli *can not functionally express the P450 enzymes that widely participate in the taxol biosynthetic pathway. Hence, *S. cerevisiae *was chosen as an alternative, since it has been reported that yeast was successfully employed as host for the engineered, multi-step production of other (mevalonate-derived) terpenoids, including steroids and carotenoids [23].

**Figure 2:**
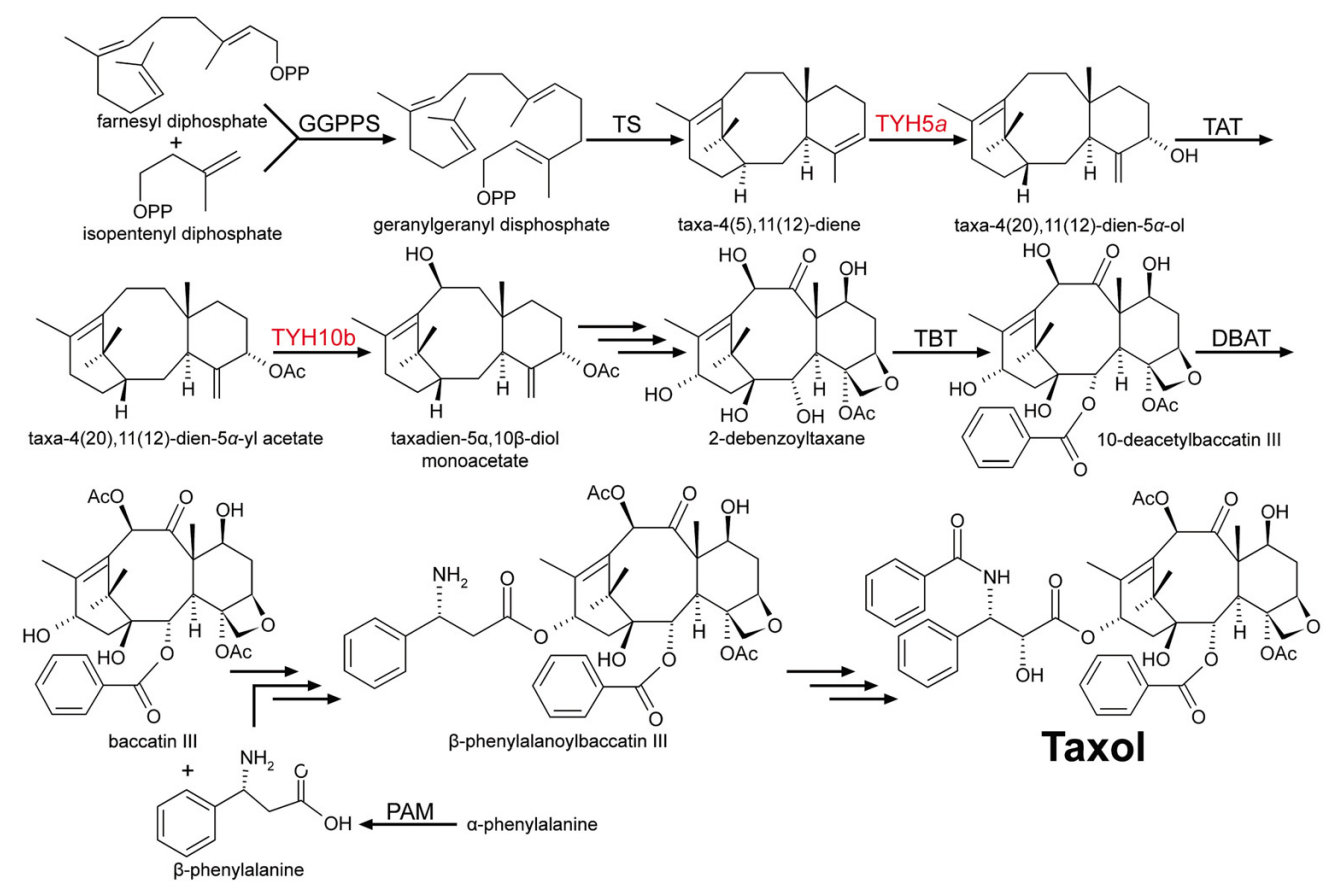
**Taxol biosynthesis pathway**. GGPPS: Genranylgenranyl diphosphate synthase; TS: taxadiene synthase; TYHa: taxadiene 5α-hydroxylase; TAT: taxa-4(20), 11(12)-dien-5a-ol-O-acetyltransferase; TYHb: taxane 10β-hydroxylase; TBT: taxane 2a-O-benzoyltransferase; DBAT: 10-deacetyl baccatin III-O-acetyltransferase. PAM: phenyalanine aminomutase. Multiple arrows indicate several as of yet undefined steps. Red text indicates a cytochrome P450 enzyme.

Recently, partial taxol biosynthetic pathway was constructed in *S. cerevisiae *by expressing five sequential pathway steps leading from primary isoprenoid metabolism to the intermediate taxadien-5α-acetoxy-10β-ol (Figure 2) [23]. The *Taxus cuspidate *genes the yeast host expressed included geranylgeranyl disphosphate synthase (GGPPS), taxadiene synthase (TS), cytochrome P450 taxadiene 5α-hydroxylase (TYH5a), taxadienol 5α-O-acetyl transferase (TAT) and taxoid 10β-hydroxylase (THY10b). The recombinant strain produced taxadiene at 1.0 mg/L while taxadien-5α-ol was produced in very small amounts (~25 μg/L). These results suggest that the first two enzymes (GGPPS and TS) cooperated well with each other and that the metabolic flux was reduced at the 5α-hydroxylation step, which is catalyzed by a cytochrome P450 hydroxylase [23]. It is anticipated that overexpressing *Taxus *P450 oxygenases with their corresponding P450 reductases in the yeast host would improve the overall production amounts [25].

Although the metabolic engineering of taxol biosynthesis in yeast has only begun, the application of new protein engineering techniques to improve rate-controlling enzymes appears promising. As a result, *S. cerevisiae *recombinant strains not only provide a new approach for taxol production, but also create platforms that allow the synthesis of taxol analogues and other rare taxoids for clinical evaluation [15].

#### Carotenoids

**Figure 3:**
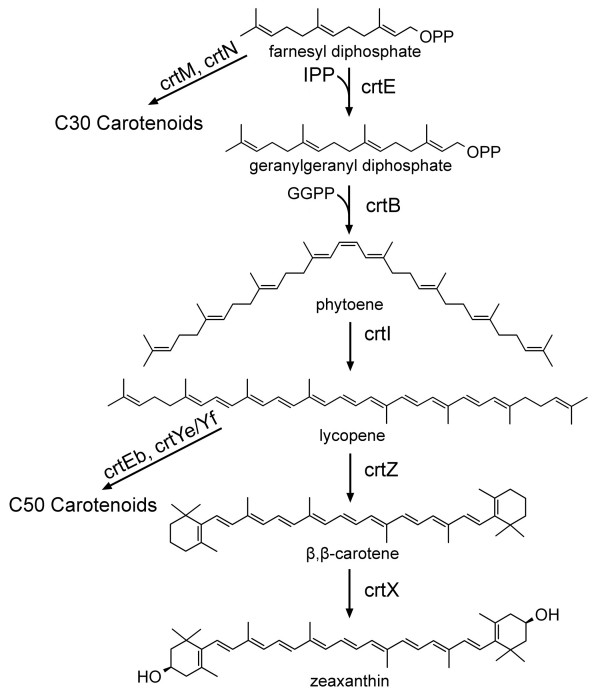
**Carotenoid biosynthesis pathway**. IPP: Isopentyl pyrophosphate; *crtE*: geranylgenranyl diphosphate synthase; *crtB*: phytoene synthase; *crtI*: phytoene desaturase; *crtZ*: β-carotene hydroxylase; *crtX*: zeaxanthin glucosylase; *crtM*: dehydrosqualene synthase; *crtN*: dehydrosqualene desaturase; *crtE*b: lycopene elongase; *crtYe/Yf*: heterodimeric decaprenoxanthin synthase.

Carotenoids are a subfamily of isoprenoids that are the most widely distributed yellow, orange and red natural pigments synthesized in bacteria, algae, fungi [26]. Commercially carotenoids such as β-carotene and astaxanthin are used as food colorants, animal feed supplements and for nutritional and cosmetic purposes. More recently, carotenoids have received attention for their significant antioxidant activities and for playing an important role in inhibiting the onset of chronic diseases [27-30]. The majority of the 600 known structures of carotenoids are C_40_-carotenoids while a few bacterial carotenoids exist with 30, 45 or 50 carbon atoms (Figure 3).

Carotenoid pathways have been successfully introduced into noncarotenogenic microbes such as *E. coli *and *S. cerevisiae *[31-35]. Since *S. cerevisiae *produces ergosterol as its principal isoprenoid from farnesyl diphosphate (FPP), it would be possible to redirect the flux of FPP away from ergosterol into genranylgenranyl diphosphate (GGPP) and subsequent carotenoids. Indeed, the insertion of a plasmid containing *Erwinia uredovora *GGPP synthase (*crtE*), phytoene synthase (*crtB*) and phytoene desaturase (*crtI*) genes under the control of various *S. cerevisiae *promoters allowed *S. cerevisiae *to produce lycopene (113 μg/g dry weight) [35]. In addition, *S. cerevisiae *harboring a plasmid containing the additional *E. uredovora *lycopene cyclase (*crtY*) gene resulted in the production of β-carotene (103 μg/g dry weight) [35].

#### Sterols

Sterols are important for the physiology of eukaryotic organisms as they form part of the cellular membrane where they modulate their fluidity and function and participate as secondary messengers in developmental signaling [36]. In yeast, ergosterol is the main sterol and is analogous to cholesterol in mammalian cells [19]. Ergosterol is also an economically important metabolite, as it is the precursor for the production of vitamin D_2_. The complex production of sterols in yeast is tightly regulated at multiple levels. One of the major bottlenecks is the reaction catalyzed by HMG-CoA (Figure 1). Overexpression of a truncated Hmg1p led to a 40-fold increase of HMG-CoA activity and resulted in an increased accumulation of squalene, an intermediate towards ergosterol, of up to 5.5% of dry matter [37]. However, there was little effect on the overall ergosterol production, suggesting additional regulation further down the pathway to ergosterol. More recently, overexpression of the post-squalene genes led to the discovery of two additional bottlenecks in the pathway, namely squalene epoxidase (Erg1p) and sterol-14alpha-demethylase (Erg11p). Overexpression of Erg1p, Erg11p and a truncated Hmg1p led to a three-fold increase of sterol content over the wild-type [38]. However, 90% of the sterol content was esterfied sterol intermediates and not ergosterol. Obviously, further regulatory mechanisms are at play preventing the quantitative accumulation of ergosterol.

Besides ergosterol, several sterol intermediates are of biological importance including methylated sterol intermediates that activate meiosis [4,4-dimethyl-5alpha-cholesta-8,14,24-trienol (FF-MAS) and 4,4-dimethyl-5alpha-cholesta-8,24-dienol (T-MAS)] [39]. FF-MAS is the substrate of Erg24p and T-MAS is the substrate of Erg25p. While supplying exogenous ergosterol or cholesterol, engineered strains with abolished heme biosynthesis and deleted *ERG25 *gene accumulated T-MAS (1.3 mg/L) while deleting both *ERG24 *and *ERG25 *genes accumulated FF-MAS (5.4 mg/L) [40]. Zymosterol is another intermediate of interest as it is the precursor of cholesterol lowering substances. By deleting sterol transmethylase (*ERG6*) and 8-carbon sterol isomerase (*ERG2*), zymosterol accumulated as the major sterol [41].

Strains able to produce sterols not native to yeast have also been engineered. For example, a single mutation of Δ^24(25)^- to Δ^24(28)^-sterol methyl transferase (SMT) enzyme from *S. cerevisiae *resulted in a protein able to synthesize mono- and bis-C24-alkylated side chains of several Δ^24(25)^- and Δ^24(28)^-sterols [42]. Also, several 24-ethyl sterols and 24-ethylidene sterols were produced in engineered *S. cerevisiae *carrying a sterol 24-methyltransferase gene from the *Arabidopsis thaliana *[43].

### Long chain Polyunsaturated Fatty Acids (PUFA) biosynthesis

**Figure 4:**
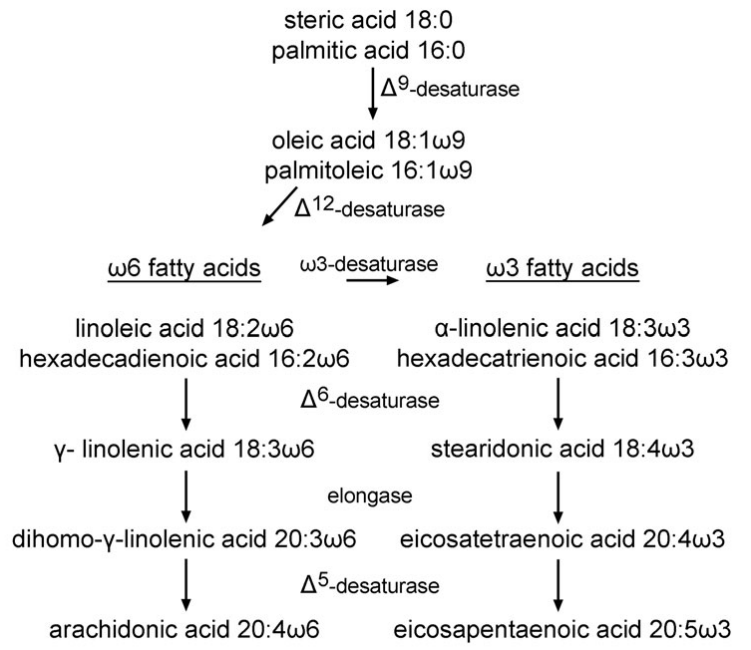
Metabolic pathways of ω3 and ω6 fatty acids.

PUFAs are fatty acids of 16 or more carbon long chains with two or more double bonds (Figure 4). Normal cellular function is dependent upon PUFAs as they are signaling molecules and regulators of membrane fluidity [44]. In general, fatty acids can be synthesized endogenously; however, PUFAs are necessary part of a diet for normal growth and development because mammals are incapable of synthesizing the essential linoleic acid (18:2ω6) [45], and ω-3 and ω-6 fatty acids. Diets rich in PUFAs were shown in studies to protect infants from cardiovascular disease, and were beneficial for the development of retina and brain functions [44,46].

PUFAs are commercially obtained primarily from seeds, marine animals, microalgae and microheterotrophs. Over the past few years, progress has been made in engineering *S. cerevisiae *for fatty acid production. More specifically, in *S. cerevisiae*, only saturated (mainly 16:0 and 18:0) and mono-saturated (18:1ω9) fatty acids, are synthesized de novo [47,48]. Formation of PUFAs requires the expression of heterogeneous genes to introduce additional double bonds (eg. Δ^6^-, Δ^12^-desaturases) and produce enzymes for chain elongation (elongases) [18,49].

A number of desaturases from a variety of sources have been discovered and functionally expressed in *S. cerevisiae *to produce PUFAs. For example, expression of the Δ^6^-desaturase from *Mucor rouxii *in yeast resulted in an accumulation of γ-linolenic acid (18:3ω6) as 7.1% of the total fatty acids when fed with linoleic acid (18:2ω6) [50]. Introducing Δ^12^-desaturases from various sources allows for the accumulation of linoleic acid (18:2) and hexadecadienoic acid (16:2) fatty acids [51-55]. Co-expression of both a Δ^6^-desaturase and a Δ^12^-desaturase from *M. alpine *resulted in endogenous γ-linolenic acid (18:3ω6) as high as 8% of total fatty acid content [56].

The range and length of PUFAs synthesized in yeast have increased with the expression of various higher-eukaryote genes. For example in yeast, arachidonic acid and eicosapentaenoic acid can be produced by coexpression of Δ^5^- and Δ^6^-desaturases with a Δ^6^-elongase in the presence of exogenously supplied linoleic acid and linolenic acid, respectfully [57,58]. A desaturase from zebrafish (*Danio rerio*) displayed both Δ^5 ^and Δ^6^-desaturase activity expressed in *S. cerevisiae *by converting linoleic acid (18:2ω6) and α-linolenic acid (18:3ω3) into their respective Δ^6^-desaturase products, γ-linolenic acid (18:3ω6) and stearidonic acid (18:4ω3) and by converting di-homo-γ-linoleic acid (20:3ω6) and eicosatetraenoic acid (20:4ω3) to their Δ^5^-desaturase products, arachionic acid (20:4ω6) and eicosapentaenoic acid (20:5ω3) [59]. The cDNAs encoding desaturase and elongase enzymes from various marine and fresh water fish, a primary source of fatty acids continue to be characterized [60,61].

### Flavonoid biosynthesis

Flavonoids are a diverse group of plant secondary metabolites that contain a 15-carbon phenylpropanoid core, which is extensively modified by rearrangement, alkylation, oxidation and glycosylation [62]. These fascinating compounds possess extraordinary antioxidant activity and they also exhibit estrogenic, antiviral, antibacterial and anti-cancer activities [63].

**Figure 5:**
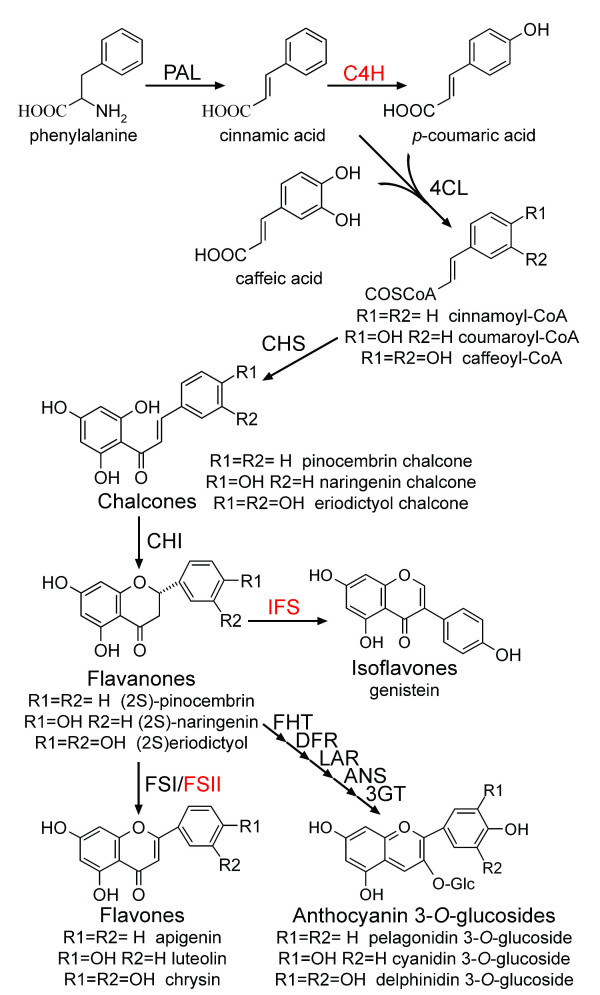
**Flavonoid biosynthesis pathway**. PAL: Phenylalanine ammonia-lyase; C4H: cinnamate-4-hydroxylase; 4CL: 4-coumaroyl:CoA-ligase; CHS: chalcone synthase; CHI: chalcone isomerase; FSI: flavone synthase; FSII: cytochrome P450 flavone synthase; IFS: cytochrome P450 isoflavone synthase; FHT: flavanone 3β-hydroxylase; DFR: dihydroflavonol 4-reductase; LAR: leucoanthocyanidin synthase; ANS: anthocyanidin synthase; 3GT: UDPG-flavonoid 3-O-glucosyl transferase. Red text indicates cytochrome P450 enzymes.

The health-protecting effects of flavonoids have stimulated significant research toward the elucidation of their biosynthetic networks (Figure 5), as well as the development of production platforms using well-characterized hosts, such as *E. coli *and *S. cerevisiae *[64]. The ability of yeast to express type II P450 hydroxylases, many of which are involved in flavonoid biosynthesis, makes it an attractive production platform, even though a recent report has demonstrated the functional expression of one such enzyme in *E. coli *[65]. Ro and Douglas [66] were the first to connect the two initial enzymes involved in phenylpropanoid pathway, namely phenylalanine ammonia lyase (PAL) and cinnamate 4-hydroxylase (C4H) in *S. cerevisiae *together with a cytochrome P450 reductase. The carbon flux through the multienzyme system from phenylalanine to *p*-coumaric acid in yeast was evaluated in their study. Later, two independent studies demonstrated the biosynthesis of flavanones, the common precursors of the vast majority of flavonoids, in *S. cerevisiae*. The first study by Jiang *et al*. [67] described the production of monohydroxylated naringenin and unhydroxylated pinocembrin at levels of 7 mg/L and 0.8 mg/L respectively. The second study by Yan *et al*. [68] reported the biosynthesis of flavanones in *S. cerevisiae *by constructing a gene cluster that included *C4H *from *A. thaliana*, *4cL-2 *from parsley, and *CHI-A *(encoding for chalcone isomerase) and *chs *from petunia. The recombinant *S. cerevisiae *strain was fed with phenylpropanoid acids and produced naringenin (28.3 mg/L), pinocembrin (16.3 mg/L) and the trihydroxylated flavanone eriodictyol (6.5 mg/L). In a continuation of the previous work, Leonard *et al *demonstrated the biosynthesis of the flavone molecules apigenin, luteolin and chrysin by exploring the expression of a soluble flavone synthase I (FSI) and a membrane bound flavone synthase II (FSII) in yeast cells. The effect of the yeast P450 reductase overexpression on flavone biosynthesis was also investigated [8]. Recently, Ralston *et al*. [69] reported the partial reconstruction of isoflavonoid biosynthesis in *S. cerevisiae *by using different types of CHI and an isoflavone synthase (IFS) from soybean (*Glycine max*).

With the rapid unraveling of the flavonoid biosynthetic pathways a wide array of flavonoid compounds, natural and unnatural, is expected to be produced from *S. cerevisiae *in the near future. In addition, the natural coloration of some of the flavonoid molecules (anthocyanins) opens up the possibility of using directed evolution for protein activity fine tuning and thus further improvement of the overall productivity [64].

## Conclusion

Ever since the era of recombinant DNA technology for natural product biosynthesis emerged [70], *S. cerevisiae *has remained the production platform of choice for many fine chemicals, including natural products. The rapid elucidation of biosynthetic pathways made possible through advanced genomic tools, has made natural product again the molecules of choice for drug development. Indeed, half of the drugs currently in clinical use are natural products and it is expected that the market size of biotech-derived small molecules will exceed 100 billion US$ in 2010 and 400 billion US$ in 2030 [71,72]. As such, we believe that *S. cerevisiae *will not only remain the cell factory of choice but that the application of powerful systems biology approaches will facilitate its expanded role in industrial applications [73-75].

## Authors' contributions

MAGK: Contributing author.

YY: Contributing author.

JAC: Contributing author and designer of all figures.
